# Supplementary material for: Overcoming evanescent field decay using 3D-tapered nanocavities for on-chip targeted molecular analysis
Source: Nat Commun. 2020 Jun 10;11:2930. doi: 10.1038/s41467-020-16813-5 (PMC7287113; doi:10.1038/s41467-020-16813-5)
Supplement: Supplementary file 1 — Supplementary Information [file 41467_2020_16813_MOESM1_ESM.pdf]

Supplementary Information

Overcoming Evanescent Field Decay Using 3D-Tapered Nanocavities for On-Chip Targeted Molecular Analysis

Kumar *et al.*

Supplementary Note 1: Device Optimization- Body

For the design purpose, the device was divided into three parts; the body, the taper, and the tip (Supplementary Figure 1a). The body width (w_{body}), height (h_{body}), and length (l_{body}) were optimized for efficient coupling of excitation light to the taper. The taper angle (α) was optimized for efficient coupling of guided AS SPP mode (Supplementary Figure 1b) to the tip as well as uniform and high electric-field ($|\mathbf{E}|^2$) enhancement for optimized emission response from molecules. The tip length (l_{tip}) was optimized for longitudinal confinement of light for detection of targeted number of molecules. The tip width (w_{tip}) and height (h_{tip}) were set to 20 nm and 50 nm, respectively. w_{tip}= 20 nm enabled controlled assembly of 1D-array antibodies (~15 nm diameter). h_{tip} was determined by adjusting the thickness of the evaporated Au layer during fabrication, and it was twice the skin depth of Au (~25 nm) or 50 nm.

A higher contrast between the effective refractive index inside the channel (n_{eff}) and the refractive index of the substrate (n_{SiO2} = 1.45) results in a higher tail-end coupling efficiency; the smaller the w_{body} and h_{body} are, the larger the n_{eff} and better the tail-end coupling of excitation light into the body (Supplementary Figure 1c). However, the smaller cross-sectional dimensions also reduced the propagation length L_m (calculated based on Supplementary Equation 1) of the guided AS mode (Supplementary Figure 1d), which led to inefficient coupling of the tail-end excitation to the taper as the guided mode experienced significant loss throughout propagation inside the body. The body with w_{body} ~ 150 nm and $h_{body} \geq$ 150 nm showed the best coupling efficiency to the taper for a l_{body} = 3 μm device (Supplementary Figure 1e). Therefore, both the w_{body} and h_{body} were set to 150 nm, which resulted in n_{eff} = 1.67 (>n_{SiO2} = 1.45), L_m = 3 μm, and the 1.12% tail-end coupling efficiency to the taper. l_{body} was set to 3 μm, which is similar to the propagation length of the guided mode, to efficiently couple the tail-end excitation light into the taper. In addition to tail-end excitation, the open-top channel of this device enables excitation of the guided mode throughout the body, which will be discussed in Supplementary Note 2.

$$L_m = 1/(2\mathrm{Im}(\mathbf{k}_m)) \qquad \text{Supplementary Equation 1}$$

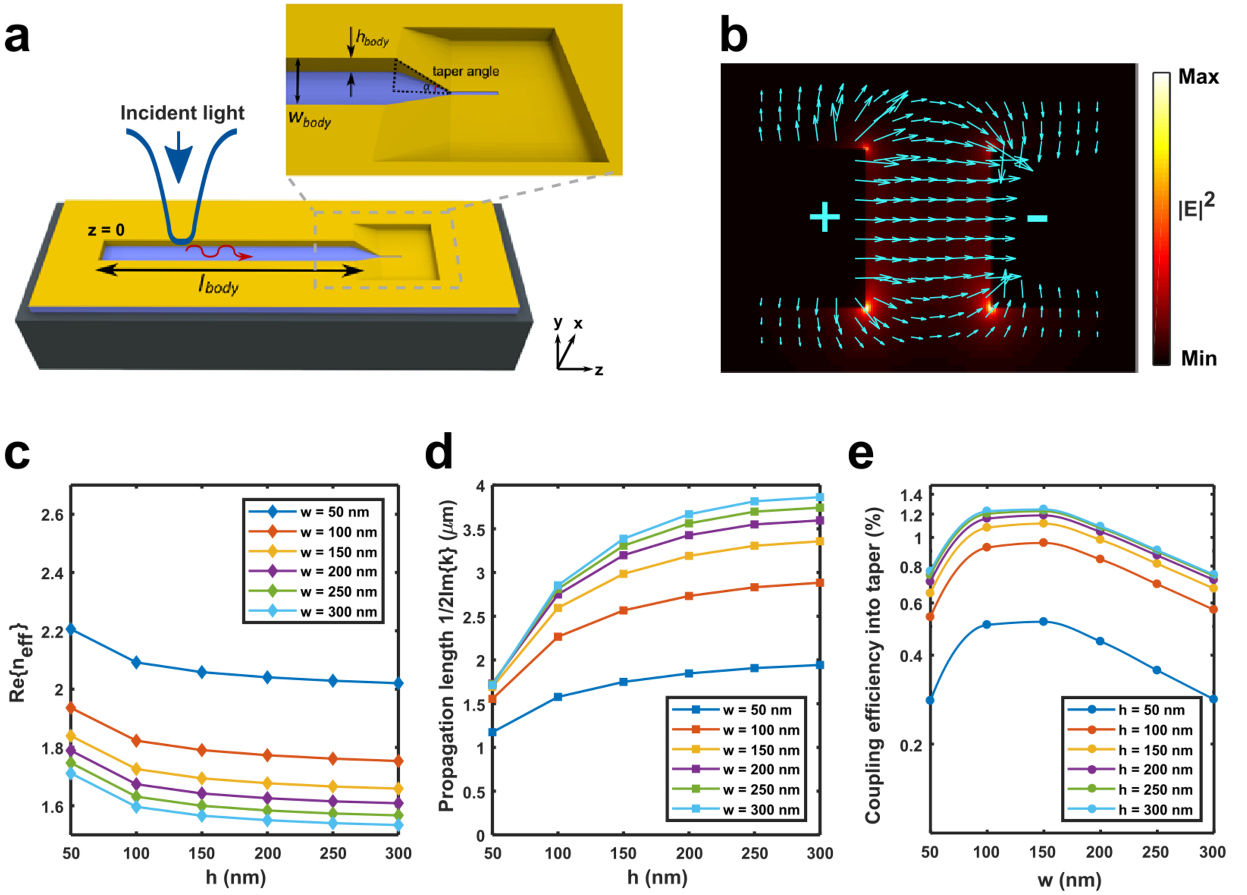

Supplementary Figure 1: Optimization of the body width and height a Schematic showing 3D-tapered nanocavity dimensions represented by symbols used in manuscript. Length of the body is l_{body}, height is h_{body}, and the taper angle is represented as α. **b** Electric-field profile of the fundamental anti-symmetric (AS) mode at the cross-section of the body. **c** Effective refractive index n_{eff} of the guided mode inside the body with varied w_{body} and h_{body}. As the w_{body} and h_{body} increase, n_{eff} decreases and the guided mode becomes less confined. **d** Propagation length L_{m} (Supplementary Equation 1) of the guided mode inside the body with varied w_{body} and h_{body}. As the w_{body} and h_{body} increase, the propagation length increases and provides more efficient coupling of guided modes into the taper. **e** Coupling efficiency to the taper with varied w_{body}, and h_{body} for a l_{body} = 3 μm device..

Supplementary Note 2: Device Optimization- Taper angle

The coupling efficiency (P_{tip}/P_{body}) was calculated based on the power at the cross-sections right before (P_{body}) and after (P_{tip}) the taper. $|\mathbf{E}|^2$ was calculated at the narrower end of the taper. Supplementary Figures 2a, b show the $|\mathbf{E}|^2$ and the coupling efficiency of devices with varying tip lengths ($l_{tip} = \infty$, 500 nm, 20 nm) for taper angles (α) ranging from 10° to 70° at the 750-nm wavelength. Both the $|\mathbf{E}|^2$ and the coupling efficiency showed the best performance within the range of $20° \leq \alpha \leq 30°$ for the various tip lengths. Experimental results showed the same trend as seen in Supplementary Figure 3. For small α, the taper length increases, and this results in greater absorption [4-6]. When α becomes large, the abrupt angle in the taper geometry results in the severe scattering of guided modes due to mismatch between the wave vectors inside the taper and the tip [4-6]. In addition, small fluctuations in the $|\mathbf{E}|^2$ and coupling efficiency in the range of $10° \leq \alpha \leq 70°$ are observed as impedance matching condition fluctuates with α (Supplementary Figures 2c-e); $\mathbf{Z}_{body} = \mathbf{Z}_{tip}$ at the peak locations observed in Supplementary Figures 3a, -b, where $\mathbf{Z}_{body(tip)}$ is the impedance of the body (tip). $\mathbf{Z}_{body}$ was calculated based on Supplementary Equation 2[7]. $\mathbf{Z}_{tip}$ at each α was calculated by iteration of Supplementary Equation 3[8] for 100 segments ($\mathbf{Z}_n$, $1 \leq n \leq 100$) along each taper length, where $\mathbf{Z}_n$ is the impedance of n-th segment calculated using Supplementary Equation 2, $\mathbf{Z}_{(n)in}$ is the input impedance of n segments, $\mathbf{k}_n$ is the complex propagation constant of the n-th segment, and l_n is the segment length.

$$\mathbf{Z} = \mathrm{R} - \mathrm{iX} = \frac{\mathrm{V}}{\mathrm{I}} = \frac{\int \mathrm{E}_x dx}{\int \mathrm{H}_y dy} \qquad \text{Supplementary Equation 2}$$

$$\mathbf{Z}_{(\mathrm{n})\mathrm{in}} = \mathbf{Z}_\mathrm{n} \frac{\mathbf{Z}_{(\mathrm{n}-1)\mathrm{in}} - \mathrm{i}\mathbf{Z}_\mathrm{n} \tanh(\mathbf{k}_\mathrm{n} l_\mathrm{n})}{\mathbf{Z}_\mathrm{n} - \mathrm{i}\mathbf{Z}_{(\mathrm{n}-1)\mathrm{in}} \tanh(\mathbf{k}_\mathrm{n} l_\mathrm{n})} \qquad \text{Supplementary Equation 3}$$

Supplementary Figure 2e shows the uniformity of the $|\mathbf{E}|^2$ enhancement in the range of $10° \leq \alpha \leq 70°$. The uniformity of the $|\mathbf{E}|^2$ enhancement was calculated based on a 2-D normalized $|\mathbf{E}|^2$ (setting 1 as the highest) profile at the cross-section of the tip. A smaller α showed better uniformity in the $|\mathbf{E}|^2$ enhancement, as a larger α increases scattering of guided modes[4-6].

The total transversal EM energy U_A and average transversal EM energy density $\bar{u}_A$ plotted in Figure 2b were calculated based on Supplementary Equations 4, 5. The efficient transversal confinement of EM energy inside the nanocavity allows uniform $|\mathbf{E}|^2$ enhancement; Figure 2b shows $U_{A\_body} \sim U_{A\_tip}$, which results in an order of magnitude greater $\bar{u}_A$ at the tip.

$$\mathrm{U_A} = \mathrm{U}_{\mathbf{E},\mathrm{A}} + U_{\mathbf{H},\mathrm{A}} = \int_{\mathrm{A}} \frac{1}{2}\varepsilon_0|\mathbf{E}|^2 + \int_{\mathrm{A}} \frac{1}{2}\mu_0|\mathbf{H}|^2$$ Supplementary Equation 4

$$\bar{\mathrm{u}}_{\mathrm{A}} = \mathrm{U_A}/(\text{cross} - \text{section of tip}) = (\mathrm{U}_{\mathbf{E},\mathrm{A}} + \mathrm{U}_{\mathbf{H},\mathrm{A}})/(\text{cross} - \text{section of tip})$$

Supplementary Equation 5

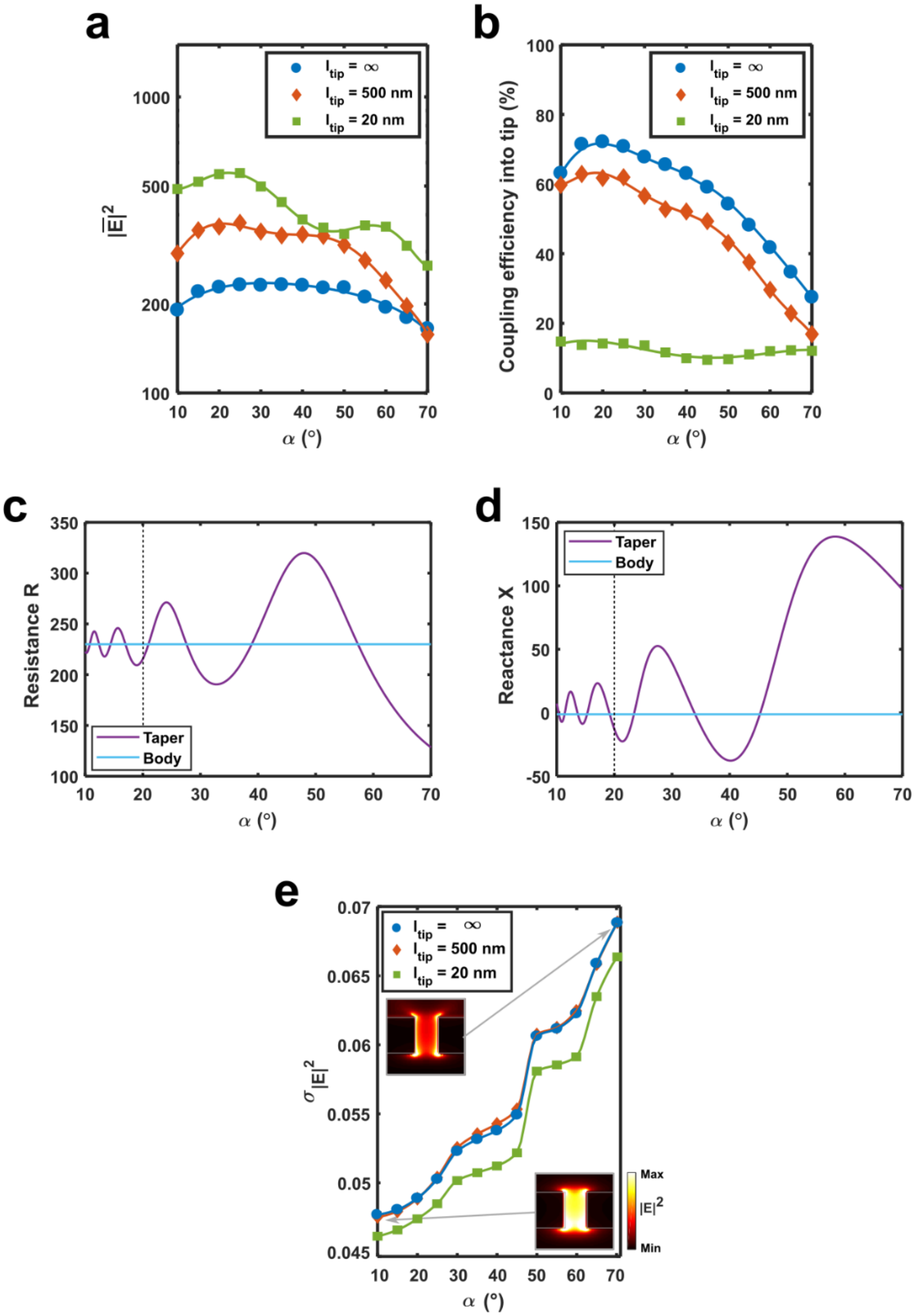

Supplementary Figure 2: Optimization of the taper angle a Average $|\mathbf{E}|^2$ enhancement ($\overline{|\mathbf{E}|^2}$) at the tip with varied taper angle α. **b** Coupling efficiency from the body to the tip with varied taper angle α. Calculated **c** resistance and **d** reactance of the taper and body based on Supplementary Equations 2,3. Impedance matching between the taper and body happens at $\alpha \sim 20°$. **e** Standard deviation of the normalized $|\mathbf{E}|^2$ enhancement distribution at the tip with varied taper angle α.

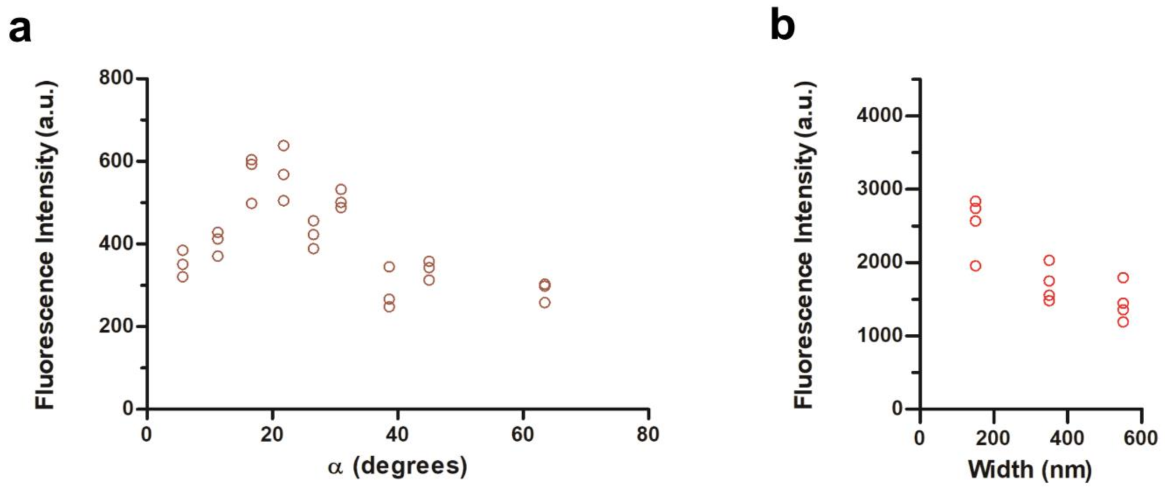

Supplementary Figure 3: Experimental device optimization a Experimental results showing that the highest fluorescence intensity from the devices was obtained for taper angle (α) close to 20°, with the body width maintained at 150 nm, tip length as 500 nm and width as 20 nm. **b** Experimental results showing that devices with body width around 150 nm had higher fluorescence emission as compared to devices with wider body widths. Device taper angle was maintained at 20° for these tests. The experimental results support the parameters indicated by simulations.

Supplementary Note 3: Device Optimization- Tip length

The length of the tip (l_{tip}) can be engineered to control the size, location, and enhancement magnitude of the hotspots formed inside the tip. A device with an infinite l_{tip} shows a gradual decrease in $|\mathbf{E}|^2$ enhancement along the tip due to absorption along the sidewalls of the tip (Supplementary Figures 4a, b). On the other hand, a device with a finite l_{tip} experiences power reflection at the end of the tip[9]. This leads to the formation of Fabry-Perot resonances of different number of peaks (m) along the longitudinal direction of the tip[10]. In this case, a properly chosen α could provide matching standing wave vectors inside the taper that could result in pronounced $|\mathbf{E}|^2$ enhancement of the resonances in the tip. In addition, a shorter l_{tip} produces intensity patterns with fewer periodic resonant peaks, which reduces the overall hotspot volume. When l_{tip} = 500 nm, the device exhibits greater $|\mathbf{E}|^2$ of the m = 4 Fabry-Perot resonance at α ~ 20° and α ~ 45° (Supplementary Figure 4a, b). When the tip is shorter (l_{tip} = 20 nm), the device shows further increase in the $|\mathbf{E}|^2$ enhancement of the m = 1 Fabry-Perot resonance at α ~ 20° and α ~ 60°. The overall $|\mathbf{E}|^2$ enhancement increases with a shorter l_{tip} due to reflection from the tip end, which increases the average volumetric EM energy density $\bar{u}_V$ inside the tip (Supplementary Figure 4c). $\bar{u}_V$ was calculated based on Supplementary Equations 6, 7. The hotspot volume shown in Figure 3f was calculated based on the full-width-half-maximum (FWHM) of each resonant peak shown in Supplementary Figure 4b.

$$U_V = U_{\mathbf{E},V} + U_{\mathbf{H},V} = \int_V \frac{1}{2}\varepsilon_0|\mathbf{E}|^2 + \int_V \frac{1}{2}\mu_0|\mathbf{H}|^2 \qquad \text{Supplementary Equation 6}$$

$$\bar{u}_V = U_V/(\text{volume of tip}) = (U_{\mathbf{E},V} + U_{\mathbf{H},V})/(\text{volume of tip}) \qquad \text{Supplementary Equation 7}$$

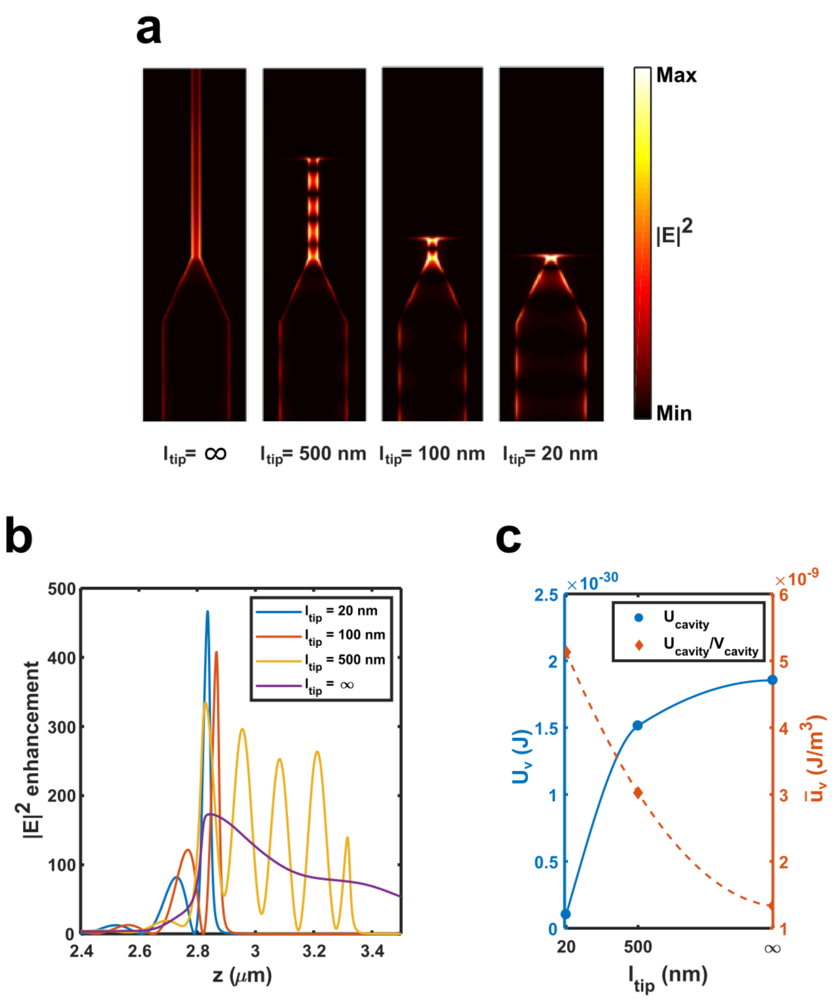

Supplementary Figure 4: **Optimization of the tip length a** Electric field distribution of the guided mode with varied tip lengths (Infinite, 500 nm, 100 nm, 20 nm, from left to right). **b** Comparison between the |**E**|² enhancement profiles of different tip lengths. A shorter tip provides higher enhancement in a more confined area. **c** Comparison between the stored energy U and average energy density ū in tips of different lengths. U and ū were calculated based on Supplementary Equation 6 and 7, respectively. A shorter tip provides a higher density of the stored energy inside the tip, whereas the total amount of stored energy decreases due to increased loss.

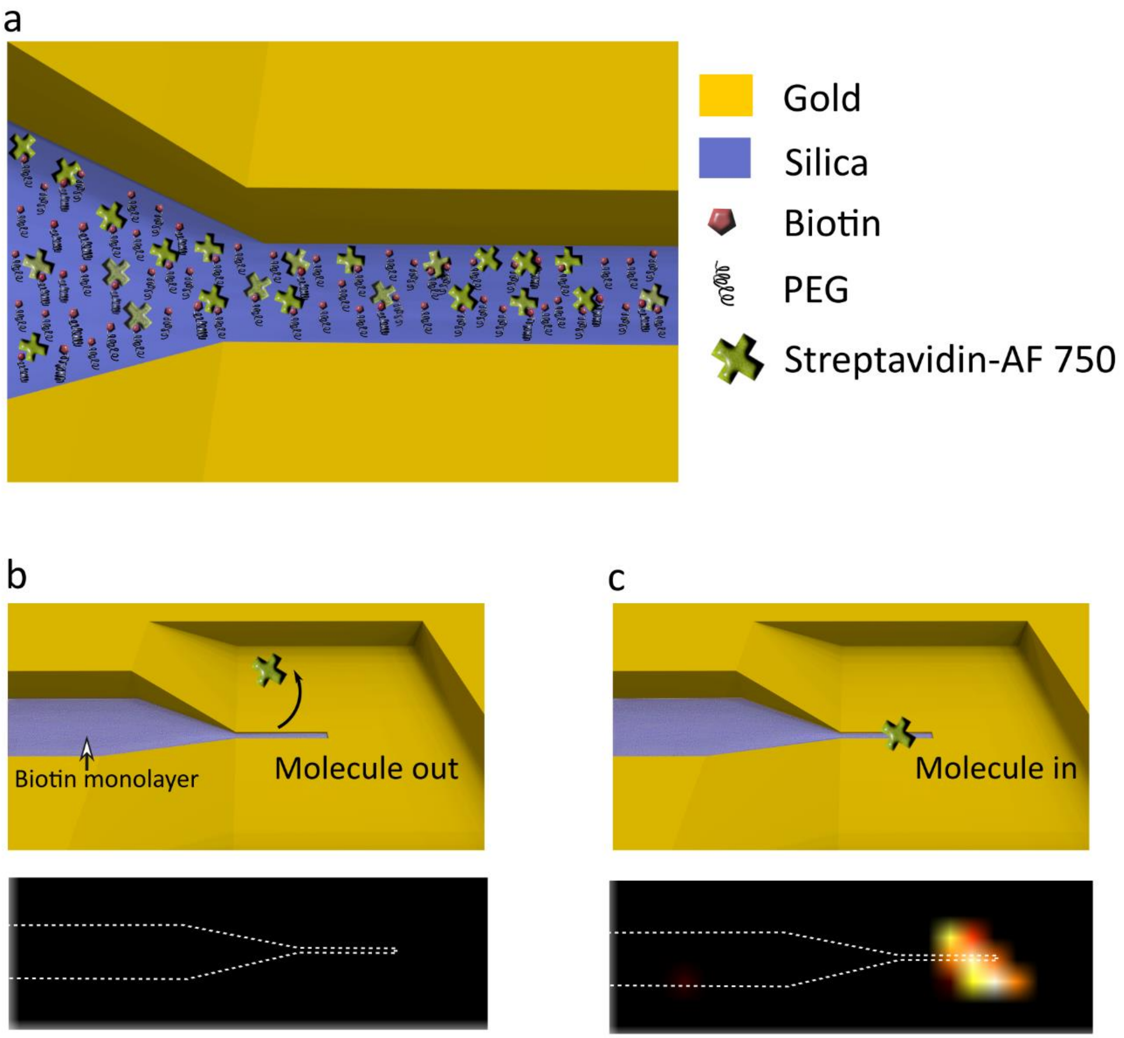

Supplementary Figure 5: Molecular functionalization and imaging a Schematic showing monolayer of PEG-biotin covering the base of the 3D-tapered nanocavity. **b** Fluorescence signal when streptavidin molecules are in solution but not at the tip. **c** Signal when a molecule binds at the tip region.

Supplementary Note 4: Two approaches to light coupling into the device

In the tail-end coupling method, light was incident only on the tail-end of the device (Figure 3a). In the full illumination method, light was incident on the full device (Figure 3a). In Supplementary Figure 6a, the coupling efficiency into the body (blue solid line) was calculated using FDTD by focusing a Gaussian light source and measuring power transmission through a cross-section at each longitudinal position of the body (z). Despite the reduced coupling efficiency into the body in the middle (0 μm < z < 3 μm), the amount of power coupled to the taper (Supplementary Figure 6b) is doubled as z ~ 3 μm due to the dramatically reduced propagation loss (Supplementary Figure 6a, red dashed line) as can be seen in Supplementary Equation 8. The total coupling efficiency to the taper under full illumination is calculated to be ~15% (Supplementary Figure 6b), which is ~10× higher than tail-end only excitation.

$$\begin{aligned}\text{Coupling efficiency into taper}\\ &= \text{Coupling efficiency into body } \times (1 - \text{Propagation loss inside body})\\ &= \text{Coupling efficiency into body} \times (1 - \mathrm{e}^{-2\alpha(3\mu\mathrm{m}-z)})\end{aligned}$$

Supplementary Equation 8

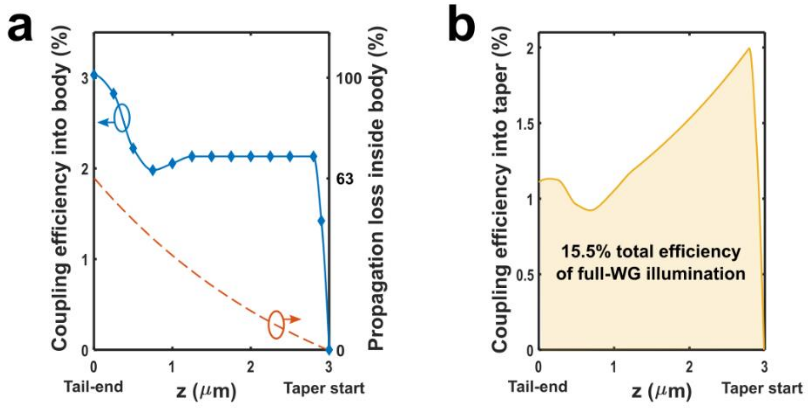

Supplementary Figure 6: Coupling efficiency of full illumination mode a Coupling efficiency into the body (solid blue line) and propagation loss of the coupled light (dashed red line) at each longitudinal location (z) along the body length. **b** Coupling efficiency to the taper at each longitudinal location calculated based on Supplementary Equation 8. Integrating the coupling efficiency along the body length, the total coupling efficiency under full-illumination is calculated to be 15.5%, which is ~10x greater than tail-end coupling efficiency shown in Supplementary Figure 1e.

Supplementary Note 5: Effect of nanocavity structure on fluorescence

Nanocavities were fabricated with the full body, just 3D taper and tip, and stand-alone tips (l_{tip} = 500 nm for all three cases) and the mean fluorescence intensity observed at the tips was measured for all the cases (Supplementary Figure 7). The trend for the nanocavities indicates that maximum intensity was obtained for the 3D-tapered nanocavity with full body. Observed fluorescence intensity sharply decreases when the whole device is reduced to just the 3D-taper and tip and decreases further for the stand-alone tip structures (Supplementary Figure 7). These observations agree with the FDTD simulation results (Supplementary Figure 8), where scattering at the backend or the sidewalls of the body incite SPP propagation towards the tip. Drastic decrease in light intensity for a taper-tip structure as well as standalone tip can be further attributed to loss in light collected from back scattering as well as edge scattering along the open edges of the body at the working wavelength.

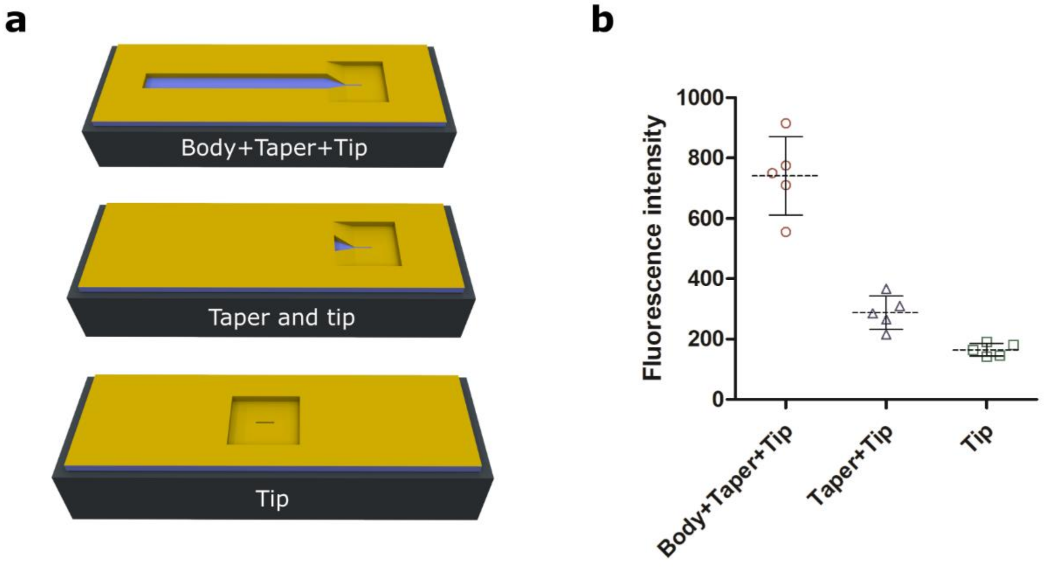

Supplementary Figure 7: Experimental study on effect of nanocavity structure on fluorescence a 3D-tapered gap plasmon nanocavity, taper and tip, and tip only structures were fabricated with fragmented body length and **b** normalized fluorescence intensities at the tip was obtained for various structures demonstrating the effect of improved coupling through the device body. Data points, mean and s.d. for 5 samples at each condition are shown.

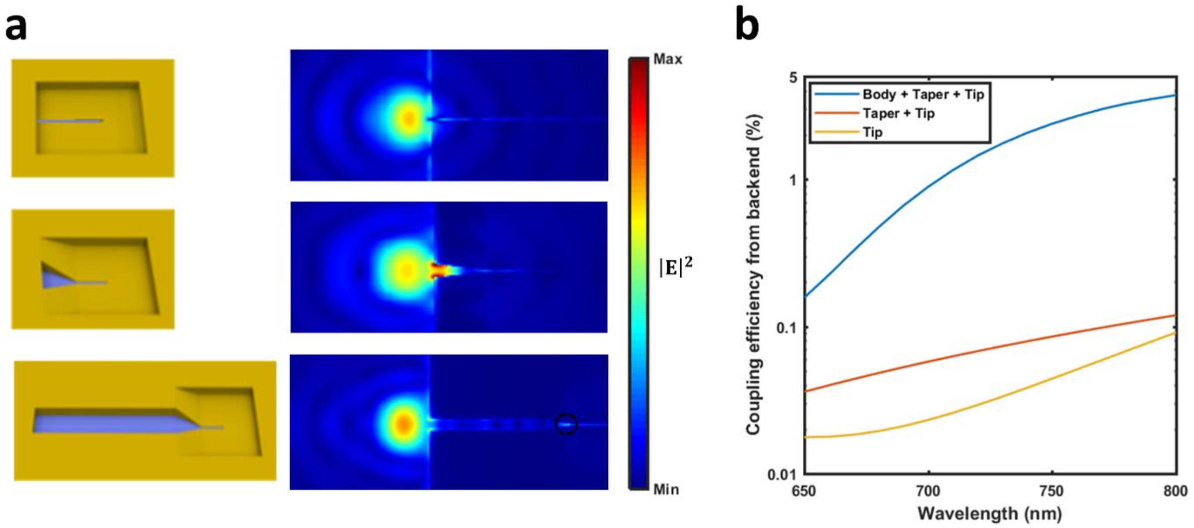

Supplementary Figure 8: Computational study on effect of nanocavity structure on fluorescence a Full waveguide, taper and tip, and tip only structures were simulated. $|\mathbf{E}|^2$ distribution at the tail-end of a tip (top), a taper and a tip (middle), and a 3D-tapered gap plasmon nanocavity (bottom). A Gaussian beam centered at 750 nm with a spot size of 270-nm diameter (obtained using a 1.4-NA objective lens) was incident at the tail-end of each structure. 3D-tapered gap plasmon nanocavity (bottom) shows a clear hotspot at the tip, which the upper two structures do not show. **b** Tail-end coupling efficiency of the three different combination structures in (a) plotted against wavelength (650-800 nm). Coupling efficiency is enhanced to more than an order of magnitude when a 3D-tapered gap plasmon nanocavity is implemented.

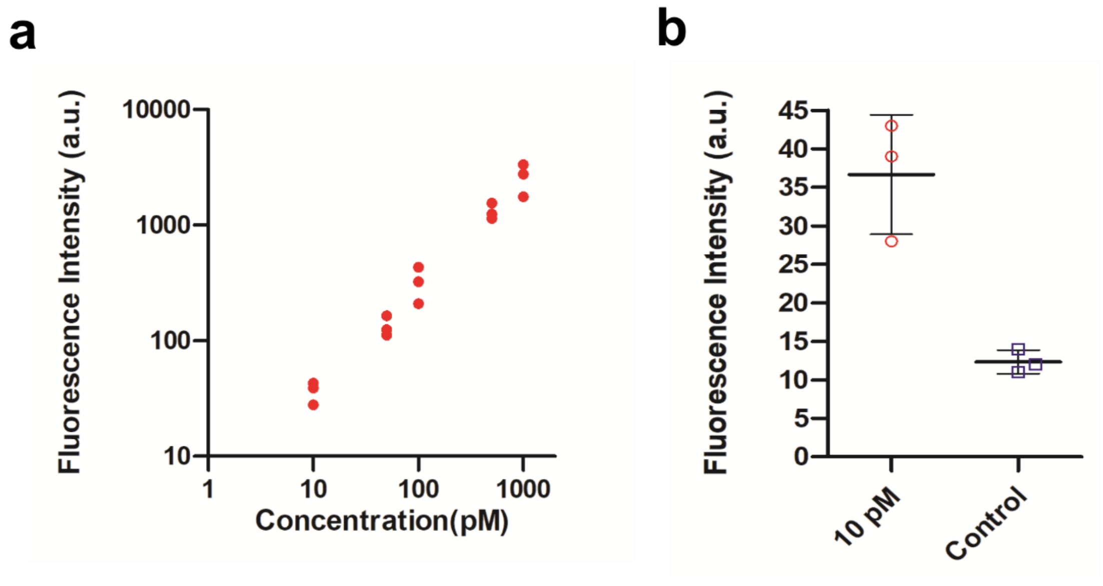

Supplementary Figure 9: Detection of low concentration molecules on 3D-tapered waveguides (tip length 500 nm) a Log-log plot showing increase in signal with increase in concentration of added Streptavidin (10 pM – 1000 pM). **b** Fluorescence signal obtained from devices after testing with 10 pM streptavidin-AF 750. Negative control device had no biotin layer. Plot shows data points, mean and s.d. for 3 devices at each condition.

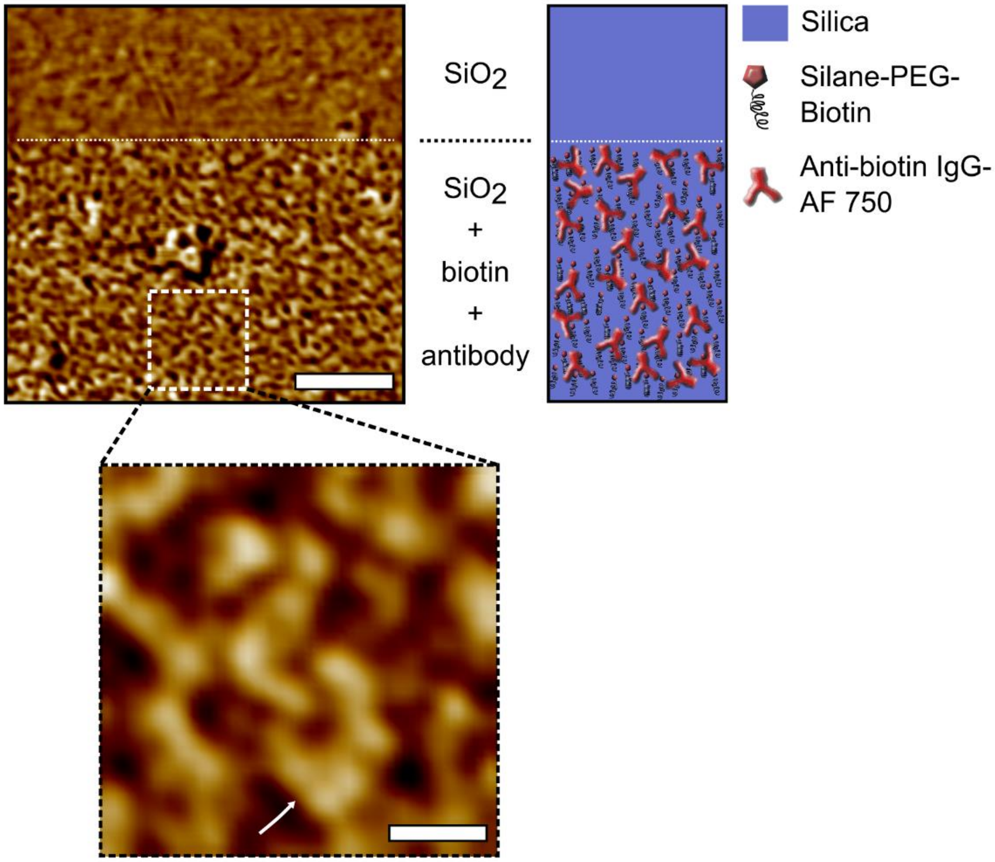

Supplementary Figure 10: Tapping-mode images of the phase signal of the PEG-biotin conjugated antibody selectively coated on silica substrate showing the uniform monolayer arrangement in large scale High resolution imaging as shown in an inset reveals the average size of the antibody of around 20 nm. The arrow highlights the typical three molecules with the tri-nodular flat orientation of the antibody. Scale bars, 200 nm (inset: 50 nm).

Supplementary Note 6: Calculation of quantum yield gain

Quantum yield gain η/η_0 of fluorophores inside the tip was calculated using FDTD. A dipole source was placed at varied locations along the x or y axis to monitor the radiation from the dipole and the tip structure. Normalized radiative decay rate γ_r/γ_0 was obtained by measuring the transmission through a closed box containing the 3D-tapered nanocavity tip structure $P_{structure}$ and dividing it by the source power P_0 (Supplementary Equation 9).

$$\frac{\gamma_r}{\gamma_0} = \frac{P_{structure}}{P_0} \qquad \text{Supplementary Equation 9}$$

Normalized non-radiative decay rate γ_{nr}/γ_0 was obtained by subtracting $P_{structure}/P_0$ from the normalized transmission through a closed box around a dipole source P_{dipole}/P_0 (Supplementary Equation 10).

$$\frac{\gamma_{nr}}{\gamma_0} = \frac{P_{dipole} - P_{structure}}{P_0} \qquad \text{Supplementary Equation 10}$$

Quantum yield gain η/η_0 was calculated using Supplementary Equation 11[11].

$$\frac{\eta}{\eta_0} = \frac{\gamma_r/\gamma_0}{1-\eta_0+\gamma_r/\gamma_0+\gamma_{nr}/\gamma_0} \times \frac{1}{\eta_0} \qquad \text{Supplementary Equation 11}$$

Supplementary Note 7: Molecular binding tests

In order to test the behavior of smaller probes, dye molecules (AF-750) were covalently linked directly to the silica-base of the nanocavities. For molecule-specific bioassays, we utilized short chain (length: 30 basepairs) single-stranded DNA aptamers labeled with AF-750 at the 5´ end. These molecules form G-quadruplex tertiary structures with dimensions ranging between 2-4 nm[12] and were used to detect insulin (monomer diameter: 2 nm[13]) that was physically adsorbed within the nanocavities[14]. As shown previously (Fig. 3b, Supplementary Figure 5), proteins (Streptavidin-AF 750, diameter: 5 nm)[15], which recognized and bound to biotinylated monolayers, (expected height: 3.5 nm)[16], were also utilized for on-chip assays. The polymeric biotin-streptavidin assembly provides an increase in probe-dimension as well as change in orientation as compared to the aptamer-based samples. Predominantly, bioassays rely on application of even larger proteins, i.e. antibodies for highly-specific detection of target molecules. IgG antibodies have a globular diameter around 15 nm, which can vary depending on the molecular mass of the antibodies and associated conjugates[17,18]. The expected average fluorophore height shown in Figure 4e includes the diameter of the surface-bound antigen (insulin or biotin) added to the diameter of the biorecognition element (aptamer, streptavidin or antibody).

Supplementary Note 8: Calculation of experimental enhancement factor

Experimental fluorescence enhancement factors were calculated by comparing the ratio of molecular fluorescence on a flat silica surface as compared to molecules placed within the tips of the 3D-tapered nanocavity structures. Using the equation[19,20]:

$$\mathrm{EF} = \frac{\mathrm{I1} \times \mathrm{N2}}{\mathrm{I2} \times \mathrm{N1}} \qquad \text{Supplementary Equation 12}$$

Where I1 is the integrated fluorescence intensity from N1 number of molecules placed inside the nanocavity tip, and I2 is the total fluorescence intensity from N2 number of molecules on a flat silica surface. Since, the labeling of the surfaces was performed using self-assembly using excess of molecules, we can assume the surfaces to have a uniform surface density of molecules, η. Then the number of molecules can be represented as: $\mathrm{N1} = \eta \times \mathrm{A1}$ and $\mathrm{N2} = \eta \times \mathrm{A2}$, where A1 is the surface area of silica within the tip and A2 is surface area of silica on the flat surface used as control. The uniform signal enhancement response shown by fluorophores attached to dimensionally-varying probes for bioassays matches well with the response predicted by simulations (Fig. 4, Supplementary Figures 11, 12).

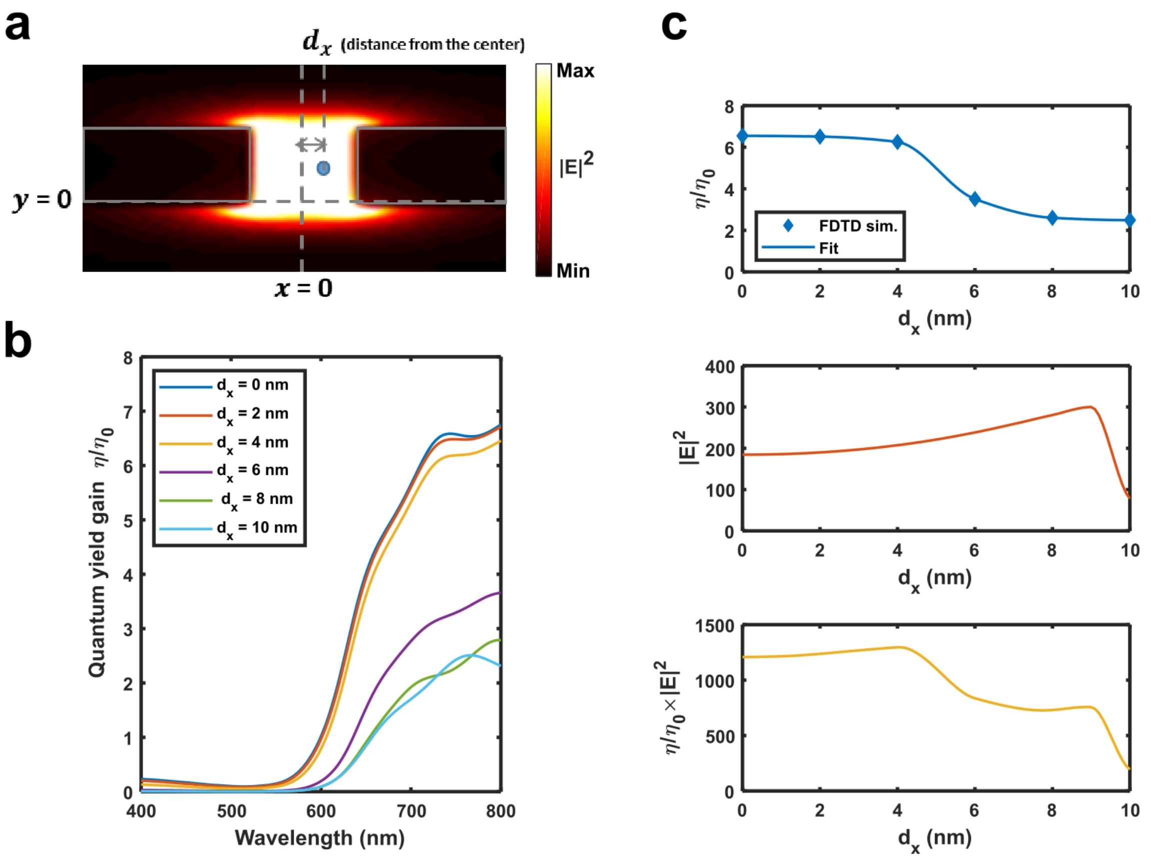

Supplementary Figure 11: Quantum yield gain of a fluorophore inside the 3D-tapered gap plasmon nanocavity along the tip width for a 500 nm long tip a Cross-sectional view of the hotspot formed at the tip. d_x is the horizontal distance of a fluorophore from the center of the tip. **b** Comparison between quantum yield gain obtained at varied horizontal locations calculated based on Supplementary Equation 11. Quantum yield gain is decreased as a fluorophore gets closer to the Au sidewall of the tip due to the quenching of fluorescent radiation. **c** Quantum yield gain η/η_0 (top), $|\mathbf{E}|^2$ enhancement (middle), and $\eta/\eta_0 \times |\mathbf{E}|^2$ (bottom) at 775 nm at varied horizontal locations.

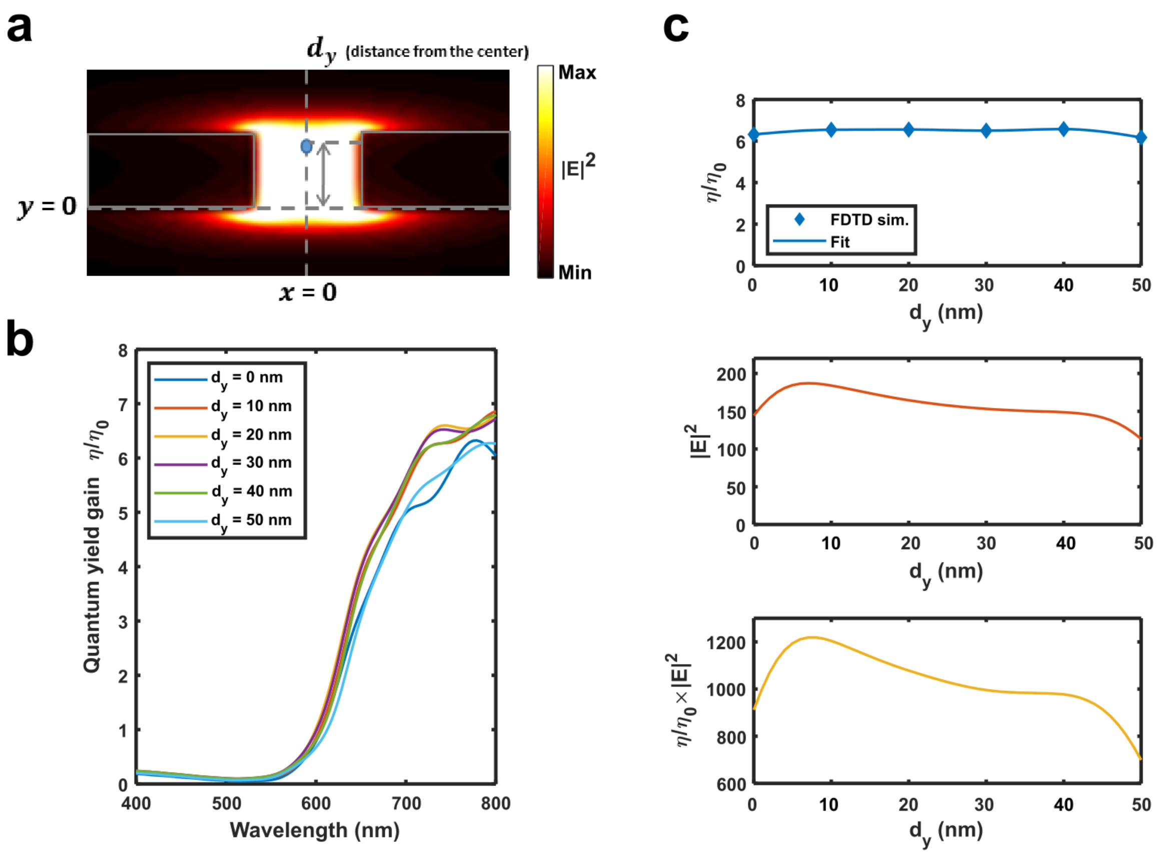

Supplementary Figure 12: Quantum yield gain of a fluorophore inside the 3D-tapered gap plasmon nanocavity along the tip height for a 500 nm long tip a Cross-sectional view of the hotspot formed at the tip. d_y is the vertical distance of a fluorophore from the substrate. **b** Comparison between the quantum yield gain at varied vertical locations based on Supplementary Equation 11. Quantum yield gain does not change significantly with varied height of fluorophores. **c** Quantum yield gain η/η_0 (top), $|\mathbf{E}|^2$ enhancement (middle), and $\eta \times |\mathbf{E}|^2$ (bottom) at 775 nm at varied vertical locations.

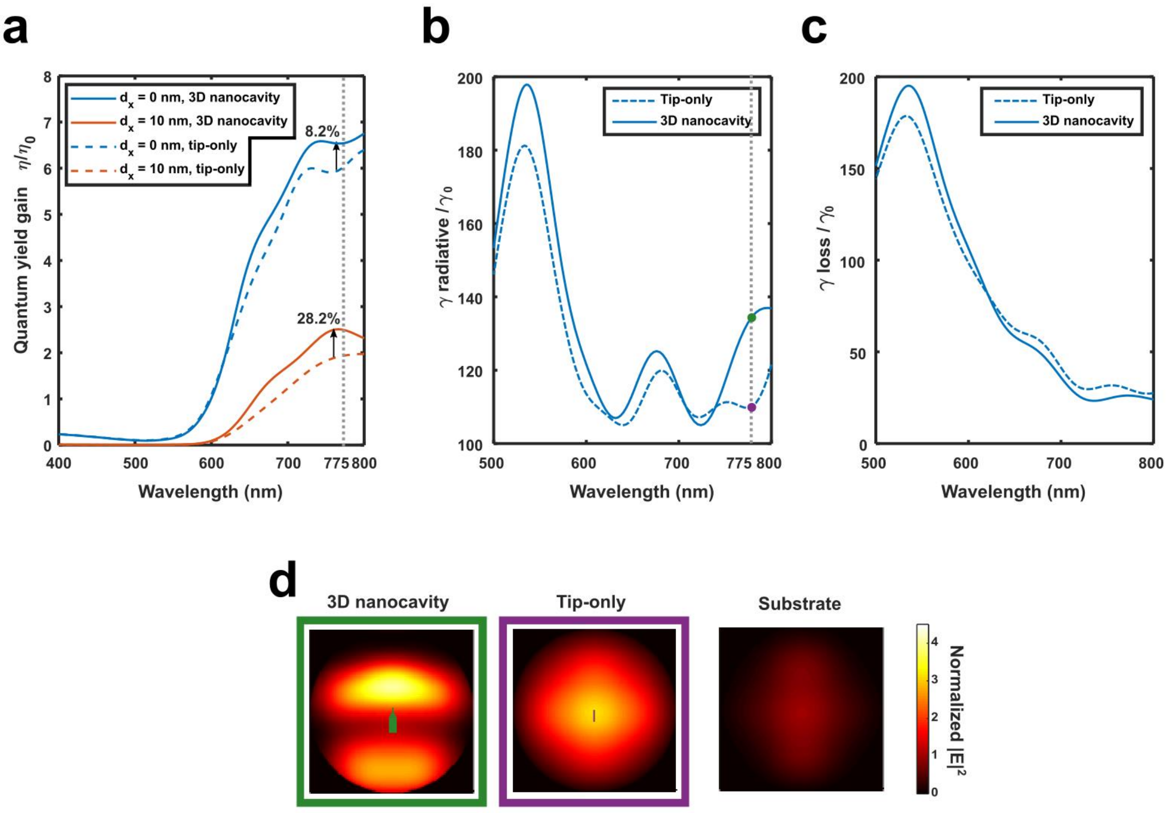

Supplementary Figure 13: Comparison between quantum yield gain profiles of a fluorophore inside the 3D-tapered gap plasmon nanocavity and tip-only structure a Quantum yield gain profiles of the 3D nanocavity (solid line) and tip-only (dashed line) structure at x = 0 (blue) and x =10 nm (red). Normalized **b** radiative decay rate and **c** non-radiative decay rate profiles of the 3D nanocavity (solid line) and tip-only structure (dashed line). **d** Normalized far-field projection of radiative intensity at 775 nm. Fluorophore was placed at x = 0, y = 25 nm inside the 3D nanocavity (green, left), inside the tip-only structure (purple, middle), and on the substrate (right). The intensity is normalized such that the far-field peak intensity from the substrate equals 1. The normalized radiative intensity peak ($\propto T_{radiative}/T_0$) represents the radiative decay rate (γ_r/γ_0) at 775 nm shown as markers in (**c**). The enhanced radiative decay rate of the 3D nanocavity results in 8.2% - 28.2% greater quantum yield gain than the conventional MIM (tip-only) structure.

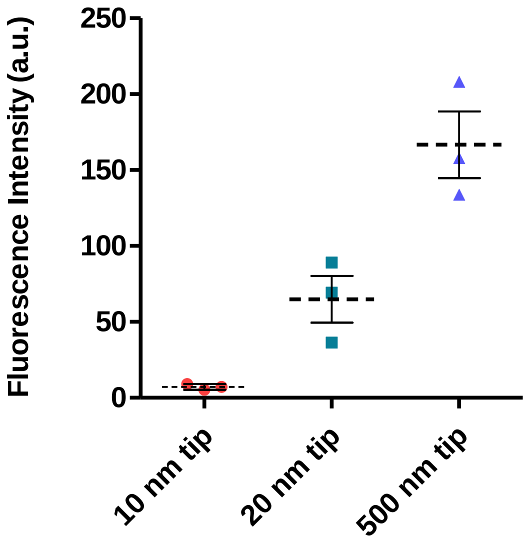

Supplementary Figure 14: Fluorescence intensity with tip length. Fluorescence intensity (background subtracted) obtained using tips of various lengths after performing binding assay with biotin and SAF-750. Individual data points, mean and s.d. for 3 devices at each condition are shown.

**Device**	**Gap size**<br>**(width of the insulator layer in MIM configuration)**	**Dye - Quantum yield (QY)**	**Reported fluorescence enhancement**<br>**(EF)**	**Fluorescence enhancement figure of merit**<br>**(EF*QY)**
Bowtie Nanoantenna[2]	20 nm	TPQDI - 0.025	1340	33.5
Antenna in box [21]	15-20 nm<br>(Fabricated using focused ion beam milling)	AF 647 with quencher - 0.08	1100	88
DNA origami nanoantenna[22]	12 nm	ATTO655 - 0.3<br>ATTO647 with quencher - 0.0034	600<br>5000	170-180
Nanocube metal film assembly[23]	10 nm	Quantum dots - 0.1	177-2300	17.7 - 230
Antenna in box[24]	10 nm<br>(Fabricated using e-beam lithography)	Crystal violet - 0.02	15000	300
**3D-tapered nanocavity**<br>***(this manuscript)**	**20 nm**	**AF 750 - 0.12**	**2200**	**264**

Supplementary Table 1: Enhancement efficiency comparison. Fluorescence enhancement and figure of merit of several previously reported nanostructures as compared to the plasmonic device presented in this manuscript. Enhancement of fluorescence is highly dependent on the quantum yield of the fluorophore used, therefore a figure of merit,[24] which normalizes the role of varying quantum yields is utilized to compare the performance of various structures. 3D-tapered nanocavity provides one of the highest enhancement figure of merits ever recorded while at the same time overcoming any variation in enhancement due to change in molecular size – which has not been demonstrated before.

Supplementary References:

1. Ko, K. D. *et al.* Nonlinear optical response from arrays of Au bowtie nanoantennas. *Nano Lett.* **11**, 61-65 (2010).
2. Kinkhabwala, A. *et al.* Large single-molecule fluorescence enhancements produced by a bowtie nanoantenna. *Nat. Photonics* **3**, 654-657 (2009).
3. Schindelin, J. *et al.* Fiji: an open-source platform for biological-image analysis. *Nat. Methods* **9**, 676 (2012).
4. Pile, D. & Gramotnev, D. K. Adiabatic and nonadiabatic nanofocusing of plasmons by tapered gap plasmon waveguides. *Appl. Phys. Lett.* **89**, 041111 (2006).
5. Conway, J. *Efficient optical coupling to the nanoscale*. Vol. 68 (2006).
6. Gramotnev, D. K., Pile, D. F., Vogel, M. W. & Zhang, X. Local electric field enhancement during nanofocusing of plasmons by a tapered gap. *Phys. Rev. B* **75**, 035431 (2007).
7. Huang, J.-S., Feichtner, T., Biagioni, P. & Hecht, B. Impedance matching and emission properties of nanoantennas in an optical nanocircuit. *Nano Lett.* **9**, 1897-1902 (2009).
8. Xu, Y., Miroshnichenko, A. E., Lan, S., Guo, Q. & Wu, L.-J. Impedance matching induce high transmission and flat response band-pass plasmonic waveguides. *Plasmonics* **6**, 337-343 (2011).
9. Choo, H. *et al.* Nanofocusing in a metal-insulator-metal gap plasmon waveguide with a three-dimensional linear taper. *Nat. Photonics* **6**, 838-844 (2012).
10. Miyazaki, H. T. & Kurokawa, Y. Squeezing visible light waves into a 3-nm-thick and 55-nm-long plasmon cavity. *Phys. Rev. Lett.* **96**, 097401 (2006).
11. Mack, D. L. *et al.* Decoupling absorption and emission processes in super-resolution localization of emitters in a plasmonic hotspot. *Nat. Comm.* **8**, 14513 (2017).
12. An, N., Fleming, A. M., Middleton, E. G. & Burrows, C. J. Single-molecule investigation of G-quadruplex folds of the human telomere sequence in a protein nanocavity. *Proc. Natl. Acad. Sci. U. S. A.* **111**, 14325-14331 (2014).
13. Blundell, T. *et al.* Three-dimensional atomic structure of insulin and its relationship to activity. *Diabetes* **21**, 492-505 (1972).
14. Yoshida, W. *et al.* Selection of DNA aptamers against insulin and construction of an aptameric enzyme subunit for insulin sensing. *Biosens. Bioelectron.* **24**, 1116-1120 (2009).

15. Hendrickson, W. A. *et al.* Crystal structure of core streptavidin determined from multiwavelength anomalous diffraction of synchrotron radiation. *Proc. Natl. Acad. Sci. U. S. A.* **86**, 2190-2194 (1989).
16. Dos Santos, N. *et al.* Influence of poly (ethylene glycol) grafting density and polymer length on liposomes: relating plasma circulation lifetimes to protein binding. *Biochim. Biophys. Acta, Biomembr.* **1768**, 1367-1377 (2007).
17. Werner, T., Bunting, J. R. & Cathou, R. E. The shape of immunoglobulin G molecules in solution. *Proc. Natl. Acad. Sci. U. S. A.* **69**, 795-799 (1972).
18. Tan, Y. H. *et al.* A nanoengineering approach for investigation and regulation of protein immobilization. *ACS Nano* **2**, 2374-2384 (2008).
19. Cai, W. *et al.* Investigation of surface-enhanced Raman scattering from platinum electrodes using a confocal Raman microscope: dependence of surface roughening pretreatment. *Surf. Sci.* **406**, 9-22 (1998).
20. Smythe, E. J., Dickey, M. D., Bao, J., Whitesides, G. M. & Capasso, F. Optical antenna arrays on a fiber facet for in situ surface-enhanced Raman scattering detection. *Nano Lett.* **9**, 1132-1138 (2009).
21. Punj, D. *et al.* A plasmonic/antenna-in-box/'platform for enhanced single-molecule analysis at micromolar concentrations. *Nat. Nanotechnol.* **8**, 512-516 (2013).
22. Puchkova, A. *et al.* DNA origami nanoantennas with over 5000-fold fluorescence enhancement and single-molecule detection at 25 μM. *Nano Lett.*, **15**, 8354-8359 (2015).
23. Hoang, T. B. *et al*. Ultrafast spontaneous emission source using plasmonic nanoantennas. *Nat. Comm.*, **6**, 7788 (2015).
24. Flauraud, V. *et al.* In-plane plasmonic antenna arrays with surface nanogaps for giant fluorescence enhancement. *Nano Lett.*, **17**, 1703-1710 (2017).
